# A Molecular Foaming and Activation Strategy to Porous N-Doped Carbon Foams for Supercapacitors and CO_2_ Capture

**DOI:** 10.1007/s40820-020-0389-3

**Published:** 2020-02-18

**Authors:** Mengyuan Zhou, Yaqian Lin, Huayao Xia, Xiangru Wei, Yan Yao, Xiaoning Wang, Zhangxiong Wu

**Affiliations:** Particle Engineering Laboratory (CPCIA) and Suzhou Key Laboratory of Green Chemical Engineering, School of Chemical and Environmental Engineering, College of Chemistry, Chemical Engineering and Materials Science, Soochow University, Suzhou, 2151213 Jiangsu People’s Republic of China

**Keywords:** Porous carbon foams, Hierarchical pore structure, Nitrogen doping, Supercapacitors, CO_2_ capture

## Abstract

Hierarchically porous carbon materials are promising for energy storage, separation and catalysis. It is desirable but fairly challenging to simultaneously create ultrahigh surface areas, large pore volumes and high N contents in these materials. Herein, we demonstrate a facile acid–base enabled in situ molecular foaming and activation strategy for the synthesis of hierarchically macro-/meso-/microporous N-doped carbon foams (HPNCFs). The key design for the synthesis is the selection of histidine (His) and potassium bicarbonate (PBC) to allow the formation of 3D foam structures by in situ foaming, the PBC/His acid–base reaction to enable a molecular mixing and subsequent a uniform chemical activation, and the stable imidazole moiety in His to sustain high N contents after carbonization. The formation mechanism of the HPNCFs is studied in detail. The prepared HPNCFs possess 3D macroporous frameworks with thin well-graphitized carbon walls, ultrahigh surface areas (up to 3200 m^2^ g^−1^), large pore volumes (up to 2.0 cm^3^ g^−1^), high micropore volumes (up to 0.67 cm^3^ g^−1^), narrowly distributed micropores and mesopores and high N contents (up to 14.6 wt%) with pyrrolic N as the predominant N site. The HPNCFs are promising for supercapacitors with high specific capacitances (185–240 F g^−1^), good rate capability and excellent stability. They are also excellent for CO_2_ capture with a high adsorption capacity (~ 4.13 mmol g^−1^), a large isosteric heat of adsorption (26.5 kJ mol^−1^) and an excellent CO_2_/N_2_ selectivity (~ 24).

**Electronic supplementary material:**

The online version of this article (10.1007/s40820-020-0389-3) contains supplementary material, which is available to authorized users.

## Highlights

- An in situ molecular foaming and activation strategy is designed and investigated for the synthesis of hierarchically porous N-doped carbon foams (HPNCFs).
- The prepared HPNCFs possess 3D macropores, uniform micropores and mesopores, ultrahigh surface areas and high N contents and show high performances in supercapacitors and CO_2_ capture.

**Electronic supplementary material:**

The online version of this article (10.1007/s40820-020-0389-3) contains supplementary material, which is available to authorized users.

## Graphical abstract

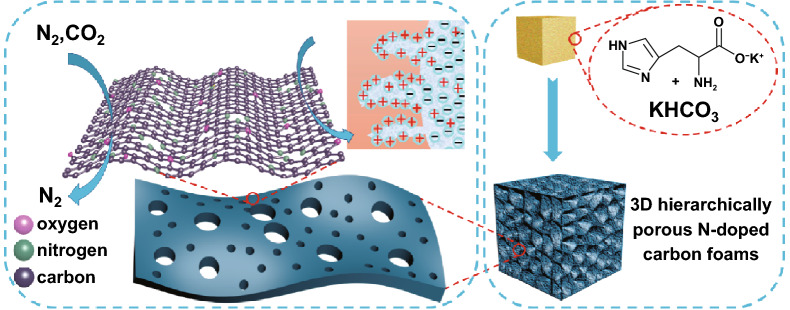

## Introduction

Porous carbon materials have received wide interest because of their attractive physicochemical properties, easy compatibility with other elements, and low cost and toxicity, as well as wide applications in energy storage and conversion, adsorption and catalysis [1–7]. A well-controlled pore structure is significantly important for these materials in various applications. Taking supercapacitors and CO_2_ capture as examples [4, 8–13], which are important for upgrading fossil fuel utilization in a greener and more sustainable way, uniform micropores and high surface areas are desirable to provide abundant active sites for enhanced storage of small-sized ions and gas molecules. However, the accessibility of micropores within thick carbon walls is low, even inaccessible for bulk molecular diffusion [4, 14, 15]. To overcome this limitation, creating mesopores in microporous carbon walls to establish short diffusion paths and large pore volumes is effective, especially so for improving charge storage at a high current density [16, 17]. In addition, a macroporous network can act as a molecule- or ion-buffering reservoir for shortening diffusion time [14]. Therefore, it is attractive to construct hierarchically macro-/meso-/microporous carbon materials (HPCs) with well-defined structures and ultrahigh surface areas to further enhance their potential.

There are a series of methods reported in the literature for the synthesis of HPCs [18]. Based on the type of precursors adopted, three general methods can be categorized. The first method is the use of preformed carbon nanomaterials, such as graphene, for the construction of low-density HPCs in the form of aerogels [19, 20]. These materials possess moderate surface areas and lack of control in the micro-/mesopore structure. The second method is carbonization of natural biomass or synthetic polymers [21, 22]. For example, carbonization of natural biomass can result in various interesting HPCs [23–34]. Control of the pore size and distribution in these HPCs is relatively difficult. In addition, their carbon walls are often relatively thick such that the accessibility of the micropores may be restricted. The third method is the templating synthesis starting from molecular precursors [35–38]. Various templates can be adopted to confine the carbonization of different precursors. In particular, the dual templating approach, in which colloid nanospheres and surfactants act as the hard and soft templates, is capable of synthesizing ordered hierarchical structures with uniform and controllable pore sizes [35, 39–42]. Nevertheless, this templating method is complicated, costly and time-consuming. Comparatively, the salt templating approach starting from molecularly mixed precursors and salts, sometimes combining with ice templating, chemical blowing and leavening, is general and cost-effective for generating various HPCs with interesting structures [43–55]. During carbonization of the precursors, the diffusion and growth of the salts or their thermal decomposition products play the templating role generating hierarchical pores. The resulted HPCs often possess broad pore size distributions with variable surface areas. In order to improve the microporosity and surface area of HPCs, post-activation with various chemical activators, especially KOH, is often adopted. A disadvantage for post-activation is the high dosage of activator and the non-uniform mixing between carbon and activator, which can subsequently result in uneven activation and excessive etching. In spite of the above development, it is still fairly challenging to simultaneously create ultrahigh surface areas, uniform micro-/mesopores and large pore volumes in HPCs.

On the other hand, doping of nitrogen (N) in porous carbon materials can increase the electronic conductivity, wettability and basicity, which is highly desirable for supercapacitor and selective CO_2_ capture [16, 56–60]. There are two major methods for N doping in carbon materials. The first method is post-treatment under high temperatures by exposing preformed carbon materials in ammonia or other N-containing substances [61, 62]. This method may cause uneven distribution of N and structure change. The more common method is in situ doping by directly pyrolysis of a single N-containing organic precursor or precursor mixture [35, 60, 63–67]. Although a series of porous N-doped carbon materials have been reported, the construction of three-dimensional HPCs with ultrahigh surface areas, large pore volumes, and high N contents sustained at high carbonization temperatures is still highly demanded.

In this work, we demonstrate an acid–base enabled in situ foaming and activation strategy for the synthesis of hierarchically macro-/meso-/microporous N-doped carbon foams (HPNCFs). Our concept for the synthesis design lies in the following aspects. First, the selected amino acid (His) as the carbon precursor shows a self-foaming behavior under heat treatment, and the selected salt PBC undergoes decomposition releasing CO_2_ to facilitate the foaming process, allowing the formation of 3D macroporous frameworks with thin walls. Second, the PBC/His acid–base reaction allows a molecular mixing for subsequent in situ uniform activation to generate narrowly distributed micropores and mesopores. Third, the imidazole moiety of His allows the sustain of a high N content after carbonization. The formation mechanism of the HPNCFs is illustrated by a detailed study. The resultant HPNCFs possess attractive properties, including 3D foam structures constructed by thin carbon walls, ultrahigh surface areas (~ 3200 m^2^ g^−1^), large pore volumes (~ 2.0 cm^3^ g^−1^), narrowly distributed micropores and mesopores and high N contents (~ 14.6 wt%). They are promising for supercapacitors showing high specific capacitances, a good rate capability and an excellent stability, as well as attractive for CO_2_ capture with a large adsorption capacity and an excellent CO_2_/N_2_ selectivity.

## Experimental Section

### Preparation of HPNCFs

The HPNCFs were prepared by a simple acid–base enabled in situ foaming and activation method. The details of the chemicals adopted for the synthesis can be found in Supporting Information. Briefly, a certain amount (0.9687–3.8748 g) of PBC was dissolved in deionized water (100 mL), and then, 2.0 g of His was added into the above solution. After completely dissolved, the obtained clear solution was transferred to an eggplant-shaped steaming bottle. The solvent was removed at 50 °C under a reduced pressure of 20–50 mbar in a rotary evaporation unit. The resulted mixture was collected in a ceramic boat and was subject to a thermal treatment in a tube furnace. The sample was heated from room temperature to various target temperatures (400–900 °C) with a heating speed of 2 °C min^−1^ and kept isothermal at the target temperature for 3 h under flowing N_2_ atmosphere (~ 60 mL min^−1^). Then, after natural cooling, the obtained composites were soaked in water at 60 °C overnight, followed by filtration, washing with deionized water and ethanol several times and drying in a vacuum oven at 60 °C for 10 h, leading to the final samples, which were denoted as HPNCF-X-Y, where X stands for the molar ratio of PBC/His, and Y for the carbonization temperature (in °C), respectively. A control sample was also prepared by carbonizing pure His without the addition of PBC following the same procedure. The characterization details of the obtained samples are provided in Supporting Information.

### Electrochemical Tests

The electrochemical tests were measured on a CHI 760e electrochemical workstation to evaluate the supercapacitor charge storage performance of the HPNCFs. In the measurement system, a Pt plate was used as the counter electrode, and a Hg/HgO electrode was served as the reference electrode. The working electrode was fabricated from the mixture of a specific HPNCF sample (80 wt%), a conductive agent (acetylene black, 10 wt%) and a binding agent PTFE (10 wt%). To prepare the working electrode, the mixture was dispersed in 2.5 mL of ethanol in an ultrasonic cleaner to form a uniform slurry and then was dried at 80 °C for 2 h to evaporate the liquid. The dried black powder was pressed onto a neat nickel foam (~ 1 × 1 cm^2^) to obtain a thin electrode membrane. The composite foam was then dried in a vacuum oven at 60 °C for 12 h. Cyclic voltammetry (CV), galvanostatic charge and discharge (GCD), electrochemical impedance spectroscopy (EIS) and cyclic tests were carried out through a three-electrode system from 0.0 to − 1.0 V with a 6.0 M KOH solution as the electrolyte. CV experiments were conducted under different sweep rates of 5–100 mV s^−1^. EIS was carried out over a frequency range of 100–0.01 Hz with a 5.0 mV AC potential amplitude. The specific capacitance (*C*_s_, F g^−1^) of the work electrode is calculated by using Eq. 1:

1 $$ C_{\text{s}} = I\Delta t/\left( {m\Delta V} \right) $$

where *I* stands for the current density (A g^−1^), Δ*t* is the discharging time (s), ΔV is the potential range in volt and *m* for the mass (g) of the active HPNCF material, respectively.

### CO_2_ Adsorption Tests

The gas adsorption performance for CO_2_ and N_2_ was evaluated by measuring the adsorption/desorption isotherms of single components and 273 and 298 K on the Micromeritics ASAP 2020 analyzer. The capacity was retrieved from the adsorption isotherms. The adsorption selectivity was calculated by using Henry’s law. The isosteric heat of adsorption (Δ*H*_ads_) was calculated by using the Clausius–Clapeyron equation (Eq. 2):

2 $$ \ln \, \left( {P_{1} /P_{2} } \right) \, = \, \left( {\Delta H_{\text{ads}} /R} \right) \times \left( {1/T_{1} {-} \, 1/T_{2} } \right) $$

where *T*_1_ and *T*_2_ (K) are the two temperatures for adsorption isotherms measurement, *P*_1_ and *P*_2_ are the pressure points on the two isotherms wherein the adsorption capacities are the same.

## Results and Discussion

### HPNCFs Formation Mechanism

**Scheme 1:**
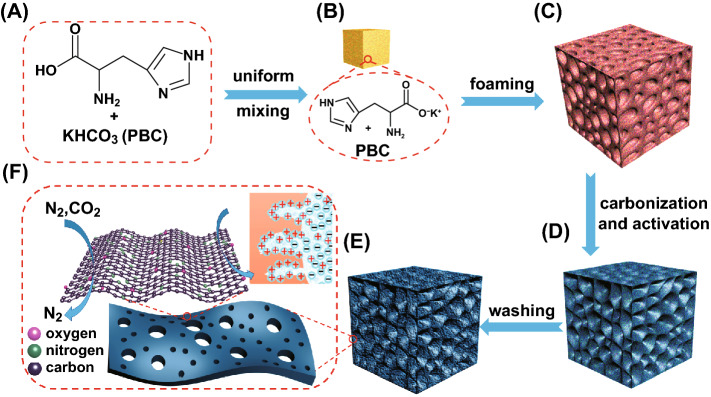
Schematic illustration of the synthesis process of the HPNCFs: **A** precursor selection, **B** the composite obtained after molecular mixing by acid–base reaction, **C** the composite obtained after in situ foaming at low temperatures, **D** the composite obtained after in situ carbonization and chemical activation at high temperatures, **E** the HPNCFs obtained after washing by water, and **F** structure model of the HPNCFs and their applications in CO_2_/N_2_ separation and charge storage

**Fig. 1:**
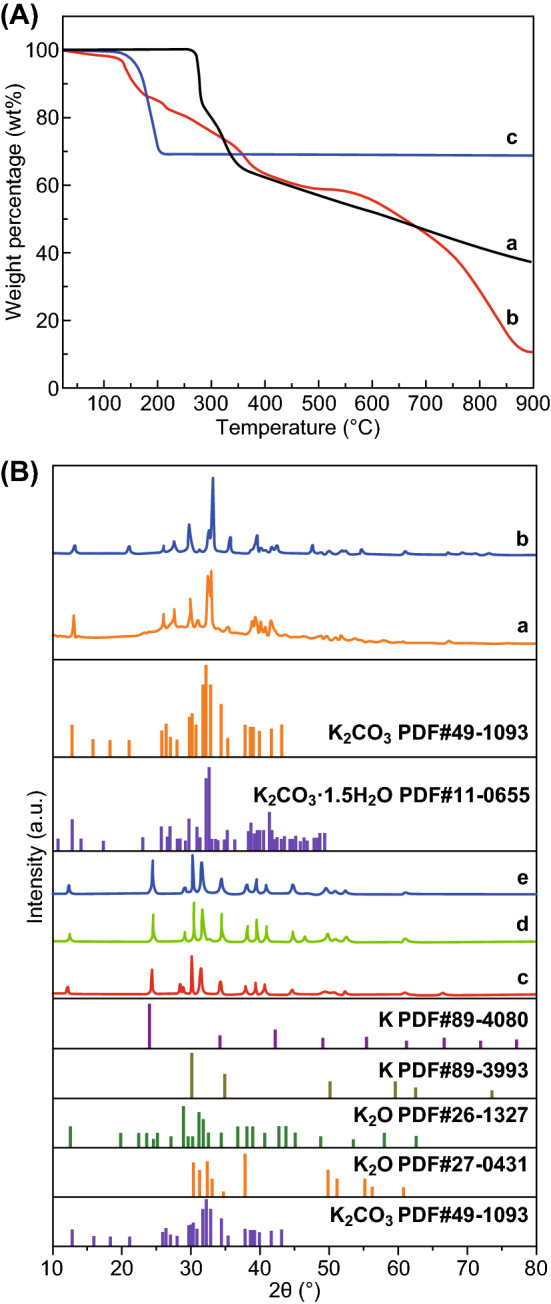
**A** TG curve under N_2_ of the pure His (a), PBC/His mixture with a molar ratio of 2.0 (b) and pure PBC (c). **B** Wide-angle XRD patterns of the composites obtained by heating the PBC/His mixture with a molar ratio of 2.0 at 400 (a), 600 (b), 700 (c), 800 (d) and 900 °C (e)

**Fig. 2:**
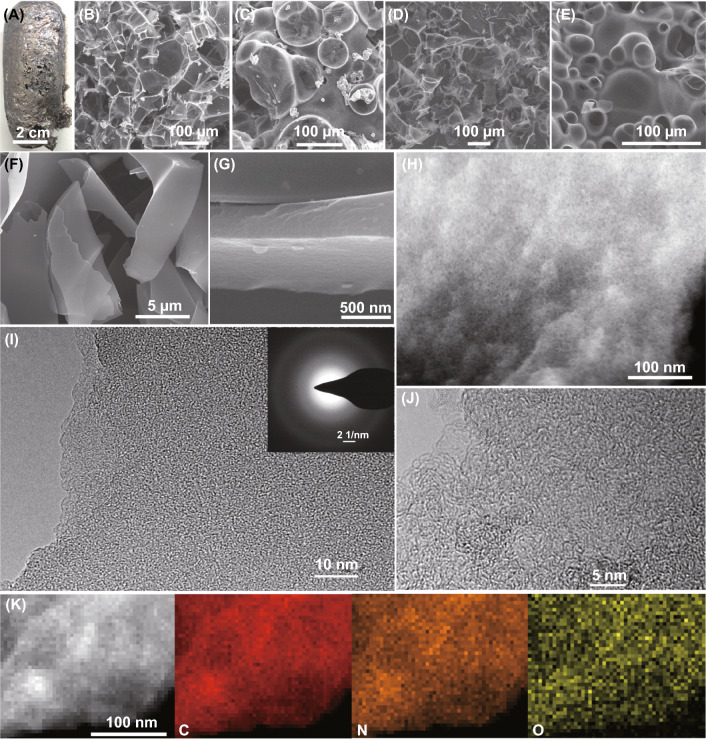
**A** Optical, **B**–**G** SEM, **H** DF-STEM, **I** TEM, inset **I** SAED pattern, **J** HRTEM, and **K** elemental maps of the HPNCF-2.0-900 sample before (**A**–**C**) and after (**D**–**K**) water washing

To synthesize the HPNCFs, an acid–base enabled in situ foaming and activation strategy has been proposed (Scheme 1). To demonstrate the formation mechanism and the unique features of resulted HPNCFs, the synthesis process with its intermediates has been analyzed. First, PBC and His can partially neutralize each other by the reaction between the bicarbonate ions of PBC and the carboxylic group of His (Eq. 3, and Scheme 1A, B). This can be validated by the decreased pH values of the mixed PBC/His solutions compared with the pure PBC solutions (Fig. S1) and the observed CO_2_ gas bubbling during mixing PBC and His in water. Such a neutralization reaction renders the molecular mixing of His and PBC (Scheme 1B), further revealed by the formation of a uniform PBC/His composite with evenly distributed C, O, N and K elements (Fig. S2). Such a molecular mixing is beneficial for in situ foaming and chemical activation. The wide-angle XRD pattern of the dried PBC/His mixture shows a group of diffraction peaks different from those of the pure PBC and His (Fig. S3), indicating the formation of a new phase probably assigned to the potassium salt of His. Next, the solid is subject to heating for in situ foaming, carbonization and chemical activation. During the heating process, sophisticated physicochemical changes are involved. Pure His is stable up to ~ 270 °C, followed by melting, polymerization and gradual carbonization at increasing temperatures (Fig. 1A, curve a). The PBC/His mixture shows a gradual mass loss at 100–270 °C (Fig. 1A, curve b), attributed to the decomposition of PBC releasing CO_2_ and H_2_O as shown in Eq. 4, indicating that the foaming probably starts at about 270 °C. The TG curve of PBC with a weight loss of ~ 30 wt% at 100–210 °C further validates the gas releasing process (Fig. 1A, curve c). This gas formation process may partially overlap with the melting of His, facilitating in situ foaming of the molten/dissolved His liquid (Scheme 1C), leading to the development of a macroporous spongy structure, similar to the leavening process [52]. The optical image after the heating treatment verifies the occurrence of foaming and the formation of a 3D macroporous foam (Fig. 2A). At 270–600 °C, the thermal behaviors of pure His and the PBC/His mixture are similar (Fig. 1A, curves a and b), indicating that chemical activation does not obviously occur at this stage. In this stage, condensation of His occurs with continuous weight loss due to the removal of water, CO_2_ and volatile N-containing molecular fragments. These released gases can lead to increased porosity in the 3D foams. The wide-angle XRD pattern of the composite obtained at 400 °C reveals the formation of a K_2_CO_3_·1.5H_2_O phase (JCPDS No. 11-0655) (Fig. 1B, curve a), indicating the decomposition of PBC. At a temperature of 600 °C, two crystalline phases assigned to K_2_CO_3_·1.5H_2_O and K_2_CO_3_ (JCPDS No. 49-1093) (Fig. 1B, curve b) can be observed. The C, N, O, and K elements are still uniformly distributed in the composite (Fig. S4a-c). The FTIR spectra of the composites obtained at 400 and 600 °C show several bands ~ 2950, 2450, 1430, 840, and 710 cm^−1^ (Fig. S5a, b), indicating the presence of K_2_CO_3_. In the temperature range of 700–900 °C, the TG curves of pure His and the PBC/His derived mixture are significantly different (Fig. 1A, curves a and b), because dramatic chemical activation occurs at this stage with the carbonaceous walls becoming increasingly thinner (Scheme 1D). At a temperature of 700–900 °C, His can be carbonized. Meanwhile, potassium oxide (K_2_O) can be formed because of the decomposition of K_2_CO_3_ and the reaction of K_2_CO_3_ with carbon as shown in Eqs. 5 and 6. The produced K_2_O and CO_2_ can in situ react with carbon as shown in Eqs. 7 and 8, generating plenty of small pores and potassium (K). The K vapor can diffuse into the carbon matrix and intercalated into graphitic layers, leading to continuous reaction with oxygen-containing carbon walls. The wide-angle XRD patterns of the composites obtained at 700–900 °C show the formation of various crystalline phases including K_2_O (JCPDS Nos. 26-1327 and 27-0431), K (JCPDS Nos. 89-3993 and 89-4080) and K_2_CO_3_ (minor) (Fig. 1B, curves c-e), confirming the occurrence of dramatic chemical activation. It should be pointed out that further decomposition of K_2_CO_3_ decomposed from pristine PBC cannot be observed at 700–900 °C for pure PBC (69 wt% remained, Fig. 1A, curve c), indicating that the presence of carbon can promote the decomposition of K_2_CO_3_ into K_2_O and K. From the PBC/His mixture with a molar ratio of 2.0, a low residual of only ~ 10.4 wt% at 900 °C confirms the formation and escape of K vapor. The elements are still uniformly distributed in the composites obtained at 700–900 °C (Fig. S4d-f), indicating that uniform chemical activation can be achieved. The FTIR spectra of the composites obtained at 700–900 °C show that the bands attributed to K_2_CO_3_ become weakened (Fig. S5c-e), in accordance with the result that K_2_CO_3_ can be converted to K_2_O and K in this temperature range. Interestingly, N_2_ sorption results show that the composites obtained at 400–900 °C possess no detectable porosity (Scheme 1D, Fig. S6a, b). This is mostly because the generated pores in the carbon walls by activation are occupied by K, K_2_O and other salts, verifying the occurrence of in situ molecular level activation. SEM images confirm the formation of a foam structure in the composite obtained at 900 °C, with the presence of a 3D macroporous structure inside and hollow carbon capsules on the surface originating from gas foaming (Fig. 2B, C). Finally, washing with water results in the HPNCFs (Scheme 1E). The foam structures are well maintained (Fig. 2D, E). Moreover, due to the presence of an imidazole moiety in His, a high N content (6.31 wt%) can be sustained even at 900 °C. The HPNCFs are suitable for supercapacitors and CO_2_ adsorption (Scheme 1F). The 3D macropores act as buffer reservoirs for guest molecules, the mesopores enhance mass transfer, and the micropores and N sites offer high specific surface areas and abundant active sites.

3 $$ {\text{C}}_{6} {\text{H}}_{9} {\text{N}}_{3} {\text{O}}_{2} + {\text{KHCO}}_{3} \to {\text{C}}_{6} {\text{H}}_{8} {\text{N}}_{3} {\text{O}}_{2} {\text{K}} + {\text{CO}}_{2} + {\text{H}}_{2} {\text{O}} $$

4 $$ 2{\text{KHCO}}_{3} \to {\text{K}}_{2} {\text{CO}}_{3} + {\text{CO}}_{2} + {\text{H}}_{2} {\text{O}} $$

5 $$ {\text{K}}_{2} {\text{CO}}_{3} \to {\text{K}}_{2} {\text{O}} + {\text{CO}}_{2} $$

6 $$ {\text{K}}_{2} {\text{CO}}_{3} + {\text{C}} \to {\text{K}}_{2} {\text{O}} + 2{\text{CO}} $$

7 $$ {\text{K}}_{2} {\text{O}} + {\text{C}} \to 2{\text{K}} + {\text{CO}} $$

8 $$ {\text{CO}}_{2} + {\text{C}} \to 2{\text{CO}} $$

### Morphology and Structure of HPNCFs

**Fig. 3:**
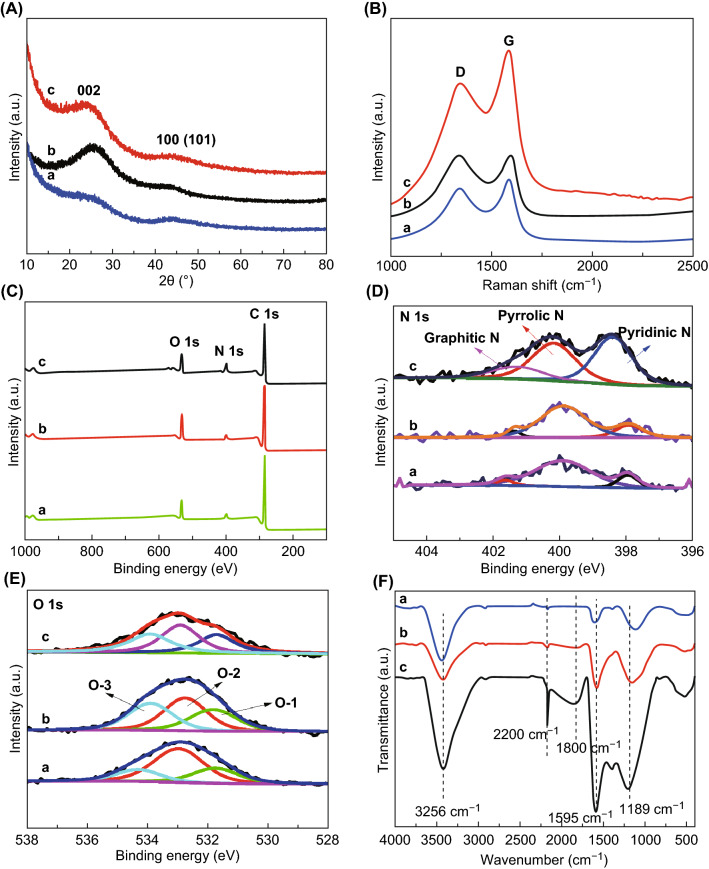
**A** Wide-angle XRD patterns, **B** Raman spectra, **C** XPS survey spectra, **D** N 1*s* XPS spectra, **E** O 1*s* XPS spectra, and **F** FTIR spectra of HPNCF-2.0-900 (a), HPNCF-2.0-800 (b), and HPNCF-2.0-700 (c)

All the HPNCFs possess a 3D hierarchically macro-/meso-/microporous structure. By using the sample HPNCF-2.0-900 obtained with a PBC/His molar ratio of 2.0 at 900 °C as a typical example, the wide-angle XRD pattern shows two weak and broad diffraction peaks at ~ 25 and 44º corresponding to (002) and (100)/(101) planes of graphitized carbon (Fig. 3A, curve a). No other peaks can be detected, implying that the K-containing substances can be washed off by water. The Raman spectrum of the sample displays two distinct bands at ~ 1341 cm^−1^ (D band) and ~ 1586 cm^−1^ (G band) (Fig. 3B, curve a). The first one can be assigned to carbon of amorphous or defective nature, and the second one to *sp*^2^ hybridized carbon of graphitic nature. The intensity ratio of the D and G bands (*I*_D_/*I*_G_) is 0.97, indicating a good graphitization degree. The sample shows a 3D foam morphology with interconnected macropores inside and some hollow carbon capsules on the surface (Fig. 2D, E). The carbon skeleton is made up of thin and smooth carbon plates (Fig. 2F). The thickness of the carbon plates is down to ~ 300 nm (Fig. 2G). Such an open 3D thin foam structure accounts for the ultralow density (about 0.03 g cm^−3^) of the sample. TEM and dark-field scanning TEM (DF-STEM) images show the presence of uniform micropores and small mesopores within the carbon plates (Fig. 2H, I), which are originating from the etching of carbon by chemical activation. The micropores are in a highly disordered orientation, and they are distributed uniformly throughout the carbon plates because of the in situ chemical activation. HRTEM image shows that very short and randomly orientated (002) graphitic layers with a d-spacing of ~ 0.37 nm can be observed (Fig. 2J). Two very weak diffraction rings indexed to the (002) and (101) crystal plate of graphitic carbon can be observed in the selected area electron diffraction (SAED) pattern (inset in Fig. 2I), further indicating that the carbon walls are moderately graphitized.

**Fig. 4:**
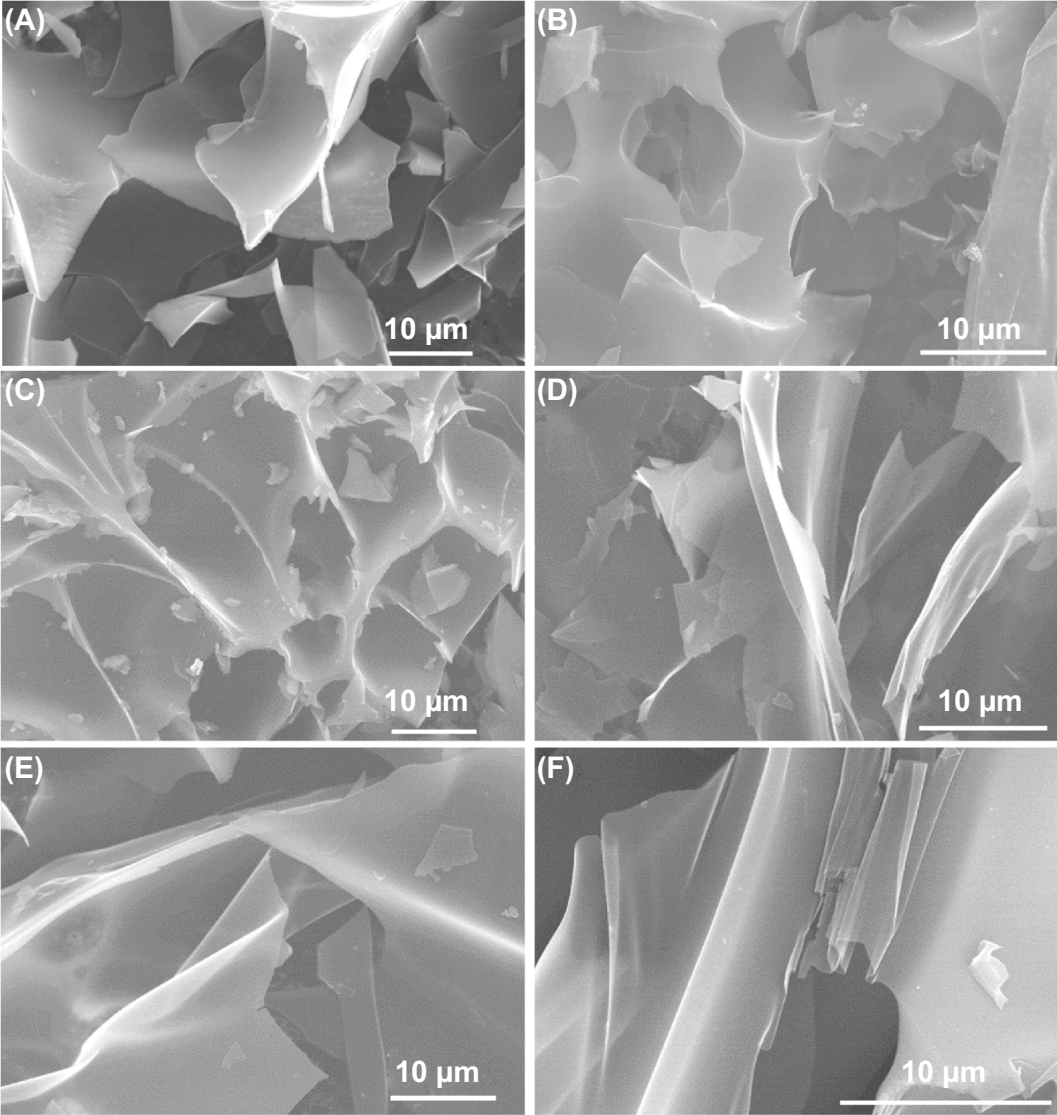
SEM images of **A** HPNCF-2.0-700, **B** HPNCF-2.0-800, **C** HPNCF-0.75-900, **D** HPNCF-1.0-900, **E** HPNCF-1.5-900, and **F** HPNCF-2.5-900

Heat treatment at 700–900 °C with a fixed PBC/His molar ratio of 2.0 does not obviously influence the overall morphology and structure of the resultant HPNCFs. An obvious difference is that the thickness of carbon plates becomes thinner with the increase in temperature (Fig. 4A, B, and 2F). This can be explained by two facts. The first one is that the carbon yield of His decreases sharply with the increase in temperature. The second one is that the chemical activation becomes increasingly violent at higher temperatures. The wide-angle XRD patterns show that the sample obtained at 800 °C possesses the best resolved (002) diffraction peak (Fig. 3A). This is because a higher temperature induces better carbonization, but also triggers more intensive chemical activation increasing structure disorder. The medium temperature of 800 °C balances the two factors leading to the most carbonized walls. The Raman spectra of the samples obtained at 700–900 °C are similar, with the lowest *I*_D_/*I*_G_ ratio observed for the sample obtained at 700 °C (Fig. 3B), which is due to the more intensive activation at higher temperatures causing loss of graphitic order.

PBC/His molar ratios of 0.75–2.5 with the fixed temperature of 900 °C also have no obvious influence on the overall morphology and structure of the resultant HPNCFs. There is also a general trend that the carbon plates become increasingly thinner with the increase in PBC dosage (Fig. 4C–F). This is because of the decreased carbon yield and the more violent chemical activation with higher PBC dosages. The wide-angle XRD patterns show that the intensity of the (002) diffraction peak decreases with the increase in the PBC/His ratio (Fig. S7A), because the enhanced chemical activation can result in increased carbon etching. The Raman spectra show a general increasing trend for the *I*_D_/*I*_G_ ratio with the increase in the PBC/His ratio (Fig. S7B), further revealing that the enhanced chemical activation reduces graphitic order.

### Textural Properties

**Fig. 5:**
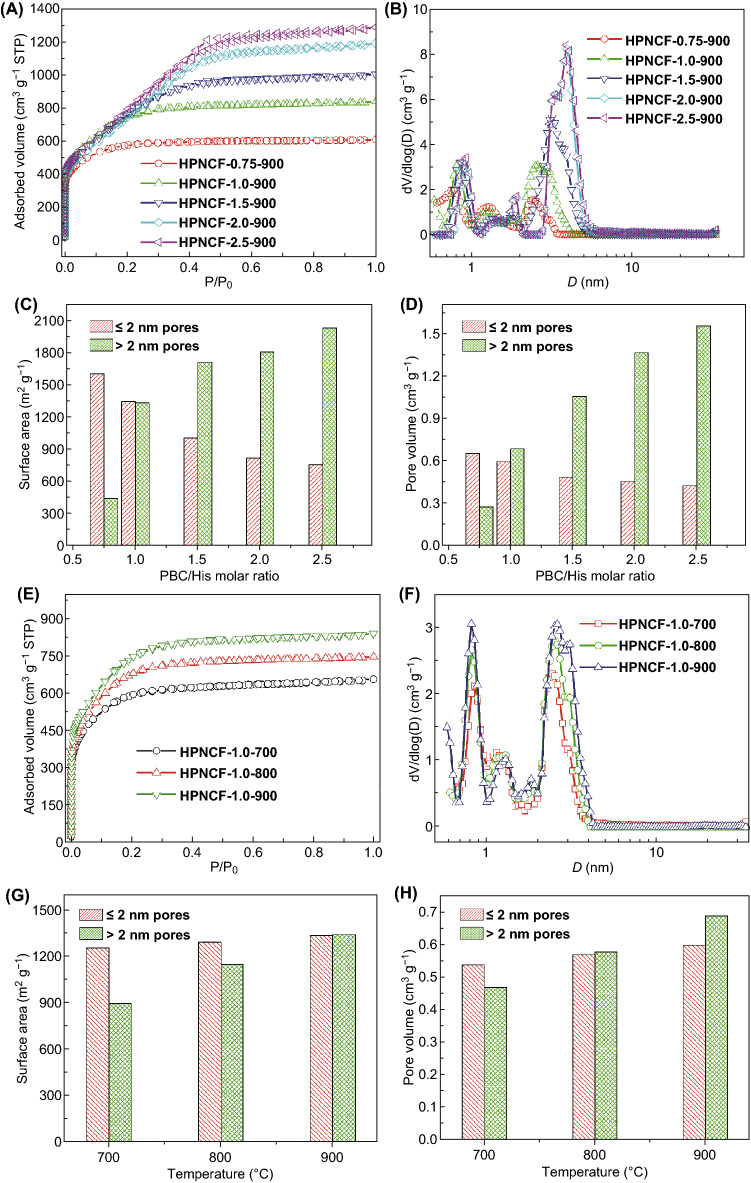
N_2_ sorption isotherms (**A**, **E**) and the PSD curves (**B**, **F**) of the HPNCFs obtained at various PBC/His molar ratios and temperatures, and the corresponding pore-size-dependent surface area (**C**, **G**) and pore volume (**D**, **H**) variations with the increase in the PBC/His molar ratio at a fixed temperature of 900 °C (**C**, **D**) and with the increase in temperature at a fixed PBC/His molar ratio of 1.0 (**G**, **H**)

All the HPNCFs possess ultrahigh surface areas, high micropore surface areas, large pore volumes and narrowly distributed micropores and small mesopores (Fig. 5 and Table 1). The representative sample HPNCF-2.0-900 displays N_2_ adsorption/desorption isotherms of combinative types I and IV (Fig. 5A), indicative of a hierarchical meso-/microporous material. Notably, at *P*/*P*_0_ < 0.1, there is a sharp and large N_2_ uptake because of the N_2_ filling in micropores. At a *P*/*P*_0_ range of 0.2–0.5, there is a N_2_ condensation step with a slight H2-type hysteresis in the desorption branch, indicating the presence of uniform and small mesopores. The sample possesses an ultrahigh specific surface area of ~ 2634 m^2^ g^−1^ and a large total pore volume of ~ 1.83 cm^3^ g^−1^. Moreover, the micropore surface area and micropore volume are up to 823 m^2^ g^−1^ and 0.46 cm^3^ g^−1^. These parameters are highly competitive compared with many HPCs in the literature (Table S1). The corresponding pore size distribution (PSD) curve of the sample reveals two narrow peaks centered at ~ 0.91 and 1.8 nm and a third peak centered at ~ 4.0 nm (Fig. 5B). In a sharp contrast, the control sample prepared from His without the addition of PBC is predominantly microporous (pore size < 0.8 nm) with a much lower surface area of 400 m^2^ g^−1^ and a significantly smaller pore volume of 0.25 cm^3^ g^−1^ (Fig. S6d). This result confirms the high efficiency of the acid–base enabled in situ chemical activation method.

**Table 1:** Summary of the textural properties and the N and O contents of the HPNCFs obtained at various PBC/His molar ratios and temperatures

Sample name	*S*_BET_ (m^2^ g^−1^)	*S*_micropore_ (m^2^ g^−1^)	*V*_total_ (cm^3^ g^−1^)	*V*_micropore_ (cm^3^ g^−1^)	Micropore (nm)	Mesopore (nm)	N (wt%)	O (wt%)
HPNCF-0.75-900	2056	1611	0.94	0.66	0.77, 1.3	2.3	3.91	7.71
HPNCF-1.0-900	2686	1348	1.29	0.60	0.82, 1.3, 1.8	2.6	4.62	11.87
HPNCF-1.5-900	2730	1011	1.55	0.49	0.86, 1.4, 1.8	3.3	5.29	9.60
HPNCF-2.0-900	2634	823	1.83	0.46	0.91, 1.4, 1.8	4.0	6.31	8.03
HPNCF-2.5-900	2793	760	1.99	0.43	0.92, 1.5, 1.9	4.0	5.90	6.12
HPNCF-1.0-700	2162	1261	1.01	0.54	0.85, 1.1	2.4	14.62	16.90
HPNCF-1.0-800	2452	1298	1.15	0.57	0.82, 1.2	2.5	9.10	14.15
HPNCF-2.0-700	3209	1381	1.66	0.67	0.88, 1.8	3.1	14.52	16.48
HPNCF-2.0-800	2305	730	1.52	0.39	0.91, 1.8	3.9	9.96	13.44

All the HPNCFs obtained at 900 °C with various PBC/His molar ratios of 0.75–2.5 possess hierarchical meso-/micropores (Fig. 5A–D and Table 1). The sample HPNCF-0.75-900 is mainly microporous showing predominant type I N_2_ adsorption/desorption isotherms (Fig. 5A). With the increase in the PBC/His molar ratio, the resultant samples are more obviously mesoporous, indicating the intensified chemical activation and carbon wall etching with the increase in PBC (Fig. 5A-D). With the PBC/His molar ratio increased from 0.75 to 2.5, the total surface area increases from 2056 to 2793 m^2^ g^−1^, and the total pore volume increases sharply from 0.94 to 1.99 cm^3^ g^−1^ (Table 1). However, the micropore surface area decreases from 1611 to 760 m^2^ g^−1^ (Fig. 5C and Table 1), and the micropore volume decreases from 0.66 to 0.43 cm^3^ g^−1^ (Fig. 5D and Table 1). On the other hand, with the increase in the PBC/His molar ratio, the PSD curves show that the mesopore size increases from 2.3 to 4.0 nm, and the micropore size also increases to some extent (Fig. 5B and Table 1). The above trends clearly confirm that the increase in PBC can enhance the chemical activation to etch more carbon walls and generate pores with increased sizes.

All the HPNCFs obtained with a fixed PBC/His molar ratio at 700–900 °C possess similar hierarchical meso-/micropores (Fig. 5E–H, S8 and Table 1). At a low PBC/His molar ratio of 1.0, there are clear increasing trends for the total surface area (2162–2686 m^2^ g^−1^), micropore surface area (1261–1348 m^2^ g^−1^), total pore volume (1.01–1.29 cm^3^ g^−1^) and micropore volume (0.54–0.60 cm^3^ g^−1^) with the temperature increased from 700 to 900 °C (Fig. 5E–H, and Table 1), while the pore size increases slightly (Fig. 5F, Table 1). Differently, at a high PBC/His molar ratio of 2.0, with the temperature increased from 700 to 900 °C, while the total pore volume (1.66–1.83 cm^3^ g^−1^) and pore size (3.1–4.0 nm) increases obviously, the total surface area (3209–2634 m^2^ g^−1^), micropore surface area (1381–823 m^2^ g^−1^) and micropore volume (0.67–0.46 cm^3^ g^−1^) decrease obviously (Fig. S8 and Table 1). Therefore, enhanced chemical activation generating more micropores can be achieved with the increase in temperature at a relatively low PBC dosage. Significant etching of carbon walls generating mesopores can be promoted with the increase in temperature at a relatively high PBC dosage.

### Chemical Composition and Surface Property

Elemental analyses reveal that all the HPNCFs are composed of C as the main component and O, N, and H as the minor ones. The DF-STEM image and the corresponding elemental maps show that the elements are evenly distributed in the sample (Fig. 2K). All the samples have high N contents (3.9–14.6 wt%) (Table 1), because the His precursor carries a theoretical N content of 22 wt% with a stable imidazole ring [66]. At a fixed PBC/His molar ratio of 2.0, with the temperate increased from 700 to 900 °C, the N content of the HPNCFs decreases from 14.52 to 6.31 wt% (Table 1), because the intensive carbonization process can break down the less stable N-containing moieties. At 900 °C, the N content of the HPNCFs generally increases (from about 4–6 wt%) with the increase in the PBC/His molar ratio (Table 1). This is probably because the etching of carbon becomes increasingly intensive with the increase in PBC, rendering relatively increased N contents. On the other hand, all the HPNCFs possess high O contents of 6.12–16.90 wt% (Table 1). With the increase in temperature, the O content decreases obviously. At the fixed temperature, there is a decreasing trend for the O content with the increase in PBC dosage. This is in agreement with the intensified carbon etching releasing CO and CO_2_ with the increase in PBC.

XPS survey spectra of the HPNCFs show three obvious bands assigned to C, N and O (Fig. 3C). The N contents estimated from the XPS analyses are in agreement with the results from element analyses. The high-resolution N 1*s* XPS spectra of the HPNCFs obtained at 700–900 °C can be well fitted into three component peaks centered at 401.4, 400.1 and 398 eV (Fig. 3D), respectively, which can be assigned to graphitic, pyrrolic and pyridinic N, respectively. Notably, with the increase in the temperature, the content of graphitic N is minimized and the content pyridinic N decreases significantly (Fig. 3D). Normally, with the increase in heating temperature, graphitic and pyridinic N are the most stable N sites in N-doped carbon materials. In the present case, the observed opposite trend is mostly because the enhanced chemical activation at higher temperatures breaks down the aromatic rings of the graphitic and pyridinic N sites. This allows a selective N-type doping in carbon materials, which will be studied in detail in our future work. On the other hand, the high-resolution O 1*s* XPS spectra (Fig. 3E) of all the HPNCFs can also be fitted into three components centered at 531.7, 532.6 and 533.7 eV, corresponding to the quinone-type oxygen (C=O, O-1), phenol-type oxygen (C–OH or C–O–C, O-2) and carboxyl-type oxygen (COO–, O-3), respectively. Among them, the O-2 band shows the highest intensity for all the samples. With the increase in the temperature, the O-2 and O-3 oxygen bands become weakened because of their relatively lower thermal stability.

The FTIR spectra of all the HPNCFs exhibit a broad absorption band at 3400 cm^−1^ with a small shoulder at ~ 3256 cm^−1^ (Fig. 3F), indicating the presence of hydroxyl groups probably from the sample surface and the adsorbed water. This peak becomes weaker with the increase in temperature. A strong sharp peak at ~ 2200 cm^−1^ and an obvious band at ~ 1800 cm^−1^ can be observed for the sample obtained at 700 °C (Fig. 3F, curve c), mostly assigned to the N=C=O and C=O moieties in the sample, and there might be some CO_2_ molecules adsorbed on the N sites contributing to the band at ~ 2200 cm^−1^. These two bands become significantly weakened in the samples obtained at higher temperatures (Fig. 3F, curves a and b), in agreement with the elemental analyses and XPS results showing dramatically decreased N and O contents. The observed broad bands at ~ 1189 and at 1595 cm^−1^ reveal the presence of benzene rings, C–O (O-2) and C–N bonding configurations. These two bands become gradually weakened with the increase in temperature because of the decreased N and O contents.

### Performance in Supercapacitors

The HPNCFs are desirable for supercapacitors. They possess ultrahigh surface areas and uniform micropores for charge storage, uniform mesopores for fast mass transfer, and 3D macropores for electrolyte storage (Scheme 1F). Moreover, their O- and N-containing groups can facilitate the infiltration and diffusion of aqueous electrolytes. These groups can also provide oxidation–reduction pathways, which can supply faradaic pseudocapacitance.

**Fig. 6:**
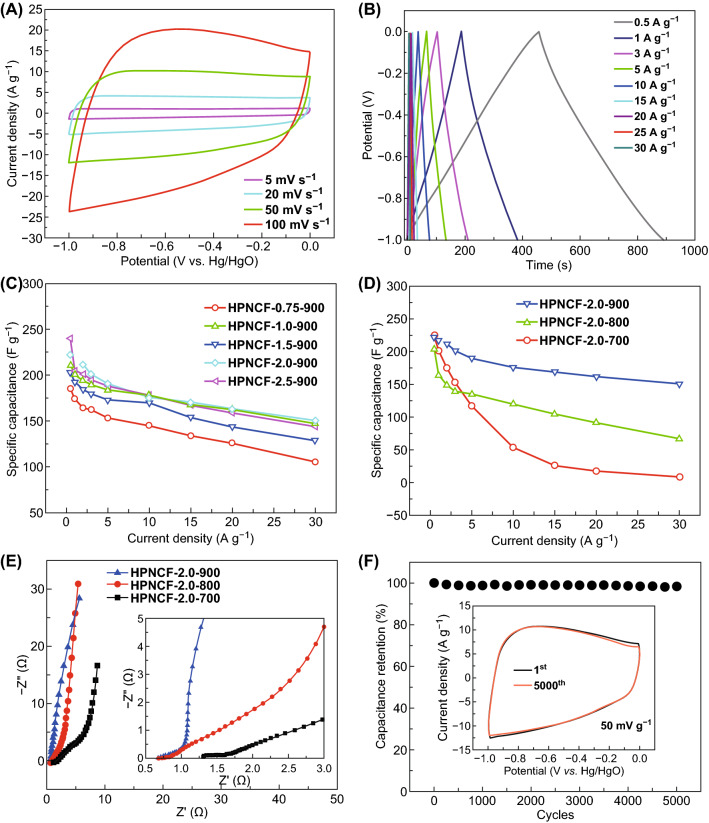
**A** CV and **B** GCD curves, and **F** cycling test of the electrode made of the sample HPNCF-2.0-900. **C** Rate capability of the HPNCFs obtained at various PBC/His molar ratios with a fixed temperature of 900 °C. **D**, **E** Rate capability and Nyquist plots of the HPNCFs obtained at various temperatures with a fixed PBC/His molar ratio of 2.0

The CV curves of the electrode made of the typical sample HPNCF-2.0-900 shows a quasi-rectangular shape over a scan rate of 5–100 mV s^−1^ in a potential window of − 1.0 to 0 V by using a 6.0-M KOH aqueous electrolyte (Fig. 6A), indicating a typical characteristic of electrical double-layer capacitance (EDLC). At the scan rate of 5 mV/s, there are small and weak redox peaks in the CV curve at ~ − 0.4 V (Fig. S9), mostly because of the redox reactions induced by the N- and O-containing groups. The GCD curves of the HPNCF-2.0-900 electrode at the current densities of 0.5–30 A g^−1^ show similar isosceles triangle shapes with no obvious IR drop (Fig. 6B), indicating a high rate capability. The specific capacitance of the HPNCF-2.0-900 sample at a current density of 0.5 A g^−1^ is ~ 222 F g^−1^. At the high current density of 30 A g^−1^, a high specific capacitance of ~ 150 F g^−1^ (~ 68% of the capacitance at 0.5 A g^−1^) can be maintained (Fig. 6C). Such performance is better than or comparable to those of many reported N-doped carbon materials (Table S1). In the Nyquist plot of the HPNCF-2.0-900 electrode, there is no visible semicircle in the high-frequency range, and a steep line nearly parallel to the vertical axis can be observed in the low-frequency region (Fig. 6E), indicative of a fast charge transfer and ionic diffusion process and a typical double-layer capacitance behavior [68, 69]. From the intercept on the X-axis, the equivalent series resistance (ESR), which reflects the electrolyte ionic resistance, the working electrode electronic resistance and contact resistance at the interface of electrode/electrolyte [70], is only ~ 0.67 Ω. To further evaluate the impedance of electrochemical system, in the equivalent circuit, *R*_s_ (the cell resistance of electrolyte and electrode) is only ~ 0.68 Ω, and *R*_ct_ (the charge transfer resistance) is only ~ 2.36 Ω (Fig. S10a), verifying that excellent charge transfer with a low resistance can be proceeded on the HPNCF-2.0-900 electrode. The rapid charge transfer is closely related to the structural features of HPNCF-2-900; that is, the 3D macroporous carbon network with hierarchal meso-/micropores can host large amount of electrolytes with a high wettability, provide abundant and accessible sites for charge storage and shorten the transfer paths of electrons and ions (Scheme 1F) [6, 28]. These features also make the HPNCF-2-900 electrode very stable. After 5000 cycles, the CV curves keep the same (inset in Fig. 6F), and only a slight capacitance loss of 1.84% can be observed (Fig. 6F).

The temperature influences the supercapacitor performance significantly of the resultant HPNCFs. The sample obtained at 800 °C shows quasi-rectangular CV curves at low scan rates, but the CV curves are much distorted at high scan rates (Fig. S11b). Distorted CV curves can be observed for the sample obtained at 700 °C at all scan rates (Fig. S11a). The specific capacitances of the samples obtained at 700–900 °C at a low current density of 0.5 A g^−1^ are close (204–225 F g^−1^) (Fig. 6D). However, the capacitance maintains only 33 and 4% for the samples obtained at 800 and 700 °C (Fig. 6D), respectively, indicative of their poor rate capabilities because of their low electronic conductivity and large electric resistance. The Nyquist plot for the sample obtained at 700 °C shows a semicircle at the high-frequency region (Fig. 6E). In the low-frequency region, the samples obtained at 800 and 700 °C show small slopes for the straight lines (Fig. 6E). The ESR is estimated to be 0.79 and 1.32 Ω for the sample obtained at 800 and 700 °C. Besides, from the equivalent circuits (Fig. S10b, c), the *R*_s_ values for the samples obtained at 800 and 700 °C are 0.80 and 1.42 Ω, and the *R*_ct_ values are up to ~ 70 and 1750 Ω, respectively. The above results reveal that the electrical resistance increases significantly for samples obtained at low temperatures, thus leading to the low rate capability.

For the HPNCFs samples obtained at various PBC/His molar ratios (0.75–2.5) at 900 °C, their specific capacitances are close (185–240 F g^−1^) at a current density of 0.5 A g^−1^ (Fig. 6C). Generally, the samples obtained at high PBC/His molar ratios possess relatively higher specific capacitances, probably due to the enhanced surface areas. On the other hand, their rate capability is similar, with 60–70% capacitance retained at 30 A g^−1^ (Fig. 6C), because these samples possess similar hierarchical macro-/meso-/micropores, as well as similar chemical compositions. These results indicate that the HPNCFs obtained at 900 °C are attractive for charge storage because of their hierarchical porosity, high surface areas and low electronic resistance.

### CO_2_ Capture Performance

**Fig. 7:**
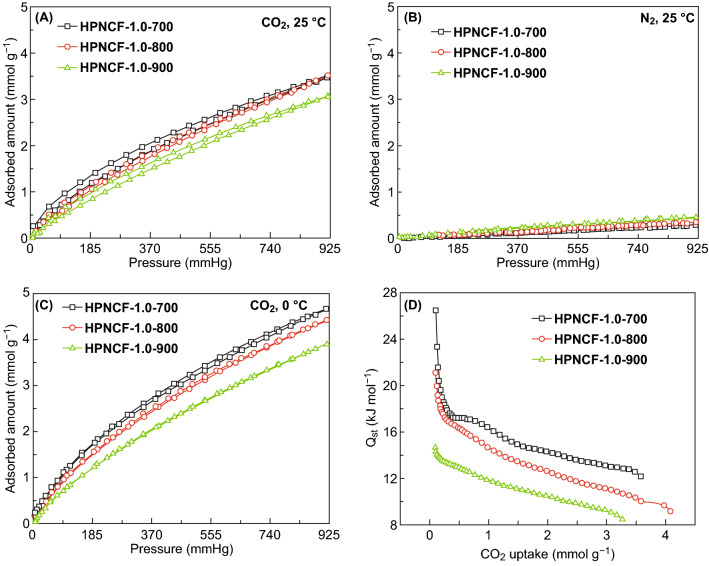
CO_2_ (**A**, **C**) and N_2_ (**B**) sorption isotherms at 25 (**A**, **B**) and 0 °C (**C**), and the corresponding isosteric heat of adsorption curves (**D**) of HPNCF-1.0-700, HPNCF-1.0-800, and HPNCF-1.0-900

The HPNCFs possess high surface areas, large micropore volumes and high N contents. These features make them promising for CO_2_ capture [71–74]. The HPNCFs obtained with a PBC/His molar ratio of 1.0 at 700–900 °C are typical for CO_2_ capture because they are mainly microporous (Table 1). The CO_2_ adsorption isotherms at 25 °C of the sample HPNCF-1.0-700 show gradual uptake of CO_2_ with the increase in pressure, and a high adsorption capacity of ~ 3.1 mmol g^−1^ can be achieved at a CO_2_ pressure of 760 torr (Fig. 7A, and Table 2). The adsorption capacity at a CO_2_ pressure of 114 torr, the normal CO_2_ partial pressure of industrial flue gas, is up to ~ 0.81 mmol g^−1^. The adsorption capacities at these two pressures can be increased to ~ 4.13 and 1.27 mmol g^−1^ at an adsorption temperature of 0 °C (Fig. 7C, and Table 2). The CO_2_ adsorption capacity of the HPNCFs decreases with the increase in the temperature from 700 to 900 °C (Fig. 7A, C), in spite of the increase in the total surface area and pore volume (Table 1). This is because CO_2_ is a small acidic molecule and its adsorption capacity is more related to the micropore surface area and the N content. The sample obtained at 700 °C possesses the highest micropore surface area and N content, thus showing the best CO_2_ adsorption capacity. Because of the strong interactions of CO_2_ molecules with the N sites and micropore walls, the sample obtained at 700 °C also shows the highest CO_2_ isosteric heat of adsorption, up to ~ 26.5 kJ mol^−1^, about two times of that for the sample obtained at 900 °C (Fig. 7D). On the other hand, the adsorption capacities of N_2_ on the above HPNCFs samples are quite low (Fig. 7B), because of the weak interactions between N_2_ and the adsorbents. As a result, an excellent CO_2_/N_2_ adsorption selectivity of ~ 24 at 25 °C on the sample obtained at 700 °C can be achieved (Table 2). The adsorption selectivity for the HPNCFs decreases with the increase in the temperature (Table 2), which is because of the much decreased N content and micropore surface area. The CO_2_ capture performance (CO_2_ adsorption capacity and CO_2_/N_2_ adsorption selectivity) of the typical HPNCFs is competitive among typical N-doped HPCs (Table S1). In addition, during cyclic tests in CO_2_ capture on the typical sample HPNCF-1.0-700 (Fig. S12), both rapid adsorption and desorption of CO_2_ processes can be observed, indicative of a predominant physisorption. After six adsorption–desorption cycles, 92% of the initial adsorption capacity can be retained, indicating a high cyclic stability for CO_2_ capture.

**Table 2:** Summary of the CO_2_ capture performance of the typical HPNCFs

Sample name	CO_2_ capacity at 25 °C		CO_2_ capacity at 0 °C		CO_2_/N_2_ selectivity at 25 °C
	114 torr	760 torr	114 torr	760 torr	
HPNCF-1.0-700	0.81	3.06	1.27	4.13	24.0
HPNCF-1.0-800	0.69	3.04	1.07	3.93	17.3
HPNCF-1.0-900	0.56	2.63	0.83	3.41	9.0

## Conclusions

In summary, novel HPNCFs have been synthesized by the acid–base enabled in situ foaming and activation strategy. The key of the strategy is the self-foaming nature of His and the CO_2_ releasing behavior of PBC allowing the formation of 3D foam structure and the acid–base reaction enabling a molecular mixing for in situ chemical activation. With the increase in temperature, His undergoes gradual carbonization with high N contents sustained. Simultaneously, phase evolutions from PBC to K_2_CO_3_·1.5H_2_O and K_2_CO_3_ and then to K_2_O and K are elucidated. The K-containing substances can in situ react with carbon walls to achieve uniform chemical activation. The HPNCFs possess 3D macroporous frameworks with thin well-graphitized carbon walls, ultrahigh surface areas (2056–3200 m^2^ g^−1^), high micropore surface areas (760–1611 m^2^ g^−1^), large pore volumes (0.94–2.0 cm^3^ g^−1^), high micropore volumes (0.39–0.67 cm^3^ g^−1^), narrowly distributed micropores (0.8–1.9 nm) and mesopores (2.3–4.0 nm), and high N contents (3.9–14.6 wt%) with pyrrolic N as the predominant N site. The increase in the PBC/His ratio results in increases in total surface area, total pore volume, pore size and N content, but decreases in micropore surface area, micropore volume and carbon wall thickness. The increase in temperature results in sharp decreases in carbon wall thickness and N content. The temperature increase induces enhancement for all the textural parameters at low PBC/His ratios, while it leads to increase in pore volume and pore size but decrease in surface area at high PBC/His ratios. The HPNCFs are promising for supercapacitors and CO_2_ capture. The HPNCFs obtained at 900 °C show high specific capacitance (185–240 F g^−1^), good rate capability and excellent stability due to the high surface area for charge storage, low electric resistance and short paths for fast electrolyte and electron transfer. The HPNCFs obtained at 700 °C show a high CO_2_ adsorption capacity (4.13 and 1.27 mmol g^−1^ at 114 and 760 torr), a large isosteric heat of adsorption (26.5 kJ mol^−1^) and an excellent CO_2_/N_2_ adsorption selectivity (~ 24). Finally, the in situ foaming and activation strategy may be extended for the synthesis of other carbon-based hierarchical structures for various applications.

## Electronic supplementary material

Below is the link to the electronic supplementary material.

Supplementary material 1 (PDF 1291 kb)
