# Supplementary material for: A Molecular Foaming and Activation Strategy to Porous N-Doped Carbon Foams for Supercapacitors and CO2 Capture
Source: Nanomicro Lett. 2020 Feb 18;12:58. doi: 10.1007/s40820-020-0389-3 (PMC7770655; doi:10.1007/s40820-020-0389-3)
Supplement: Supplementary file 1 — Supplementary material 1 (PDF 1291 kb) [file 40820_2020_389_MOESM1_ESM.pdf]

Supporting Information for

A Molecular Foaming and Activation Strategy to Porous N-Doped Carbon Foams for Supercapacitors and CO_2 Capture

Mengyuan Zhou[1], Yaqian Lin[1], Huayao Xia[1], Xiangru Wei[1], Yan Yao[1], Xiaoning Wang[1], Zhangxiong Wu[1,] *

[1]Particle Engineering Laboratory (CPCIA) and Suzhou Key Laboratory of Green Chemical Engineering, School of Chemical and Environmental Engineering, College of Chemistry, Chemical Engineering and Materials Science, Soochow University, Suzhou, Jiangsu 2151213, People's Republic of China

*Corresponding author. E-mail: zhangwu@suda.edu.cn (Zhangxiong Wu)

S1 Chemicals

The basic amino acid histidine (denoted as His) was purchased from Sigma-Aldrich. Potassium bicarbonate ($KHCO_3$, denoted as PBC) and ethanol were supplied by Sinopharm Chemical Reagent, Ltd. The Nafion solution (5.0 wt%) was purchased from DuPont. The polytetrafluoroethylene (PTFE) solution (60 wt%) was purchased from J&K. The N_2 (99.999 %) and CO_2 (99.99 %) gases were purchased from Linde Electronics Specialty Gases. All the materials were used directly without further purification.

S2 Measurement and Characterization

Scanning electron microscopy (SEM) images were obtained on Hitachi scanning electron microscopes (SU1510, S4700 and S8010), operated at 15 kV. Transmission electron microscopy (TEM) images were recorded by using FEI Tecnai G-20 and F-20 microscopes operated at 200 kV. X-ray diffraction (XRD) patterns were acquired by using a Bruker D2 Phaser X-ray diffractometer with the Cu Kα source at 30 kV and 10 mA. Raman spectra were measured by a Re Renishaw UV-1000 Photon Design spectrometer with a 532 nm excitation beam focused through a 50×microscope objective for a total interrogation spot size of ~ 1 mm. Transformed infrared (FTIR) spectra were performed on a Bruker TENSOR II Fourier transform infrared spectrometer by scanning from 4000 to 400 cm^{-1}. The measurement was conducted by mixing a certain sample and KBr and pressed into a disk. Before sample preparation, all samples were dried at 60 ºC under vacuum overnight. X-ray photoelectron spectra (XPS) were recorded on a Thermo Fisher ESCALAB 250Xi spectrometer calibrated with the C 1s at 284.6 eV. The porosity was determined by

nitrogen adsorption-desorption isotherms on a Micrometritics ASAP 2020 analyzer at -196 ºC. Before measurement, the samples were degassed at 180 ºC for at least 8.0 h to remove the moisture and impurity gases. The BET (Brunauere-Emmette-Teller) method was used to calculated the specific surface area (S_{BET}). The total pore volume (V_t) was obtained from the amount of nitrogen adsorbed under a relative pressure (P/P_0) of 0.99. The pore size distribution (PSD) curves were calculated by using the Quench Solid State Density Functional Theory (QSDFT) method. The micropore surface area (S_{mic}) and micropore volume (V_{mic}) were estimated by using the *t*-plot method. Elemental analyses (EA) were measured on a varioEL analyzer using the CHN model.

S3 Supplementary Figures and Tables

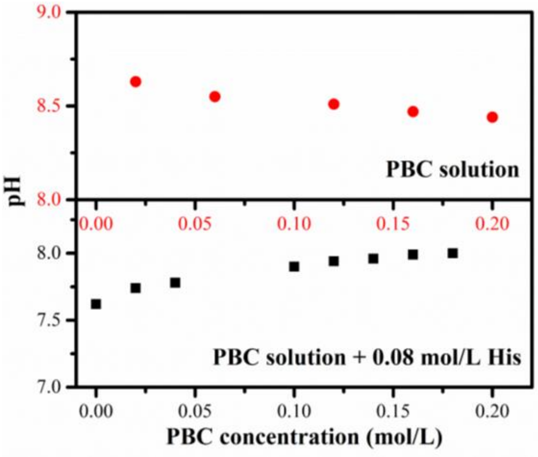

Fig. S1 Variations of the pH values for the pure PBC and PBC/His mixed solutions at different PBC concentrations

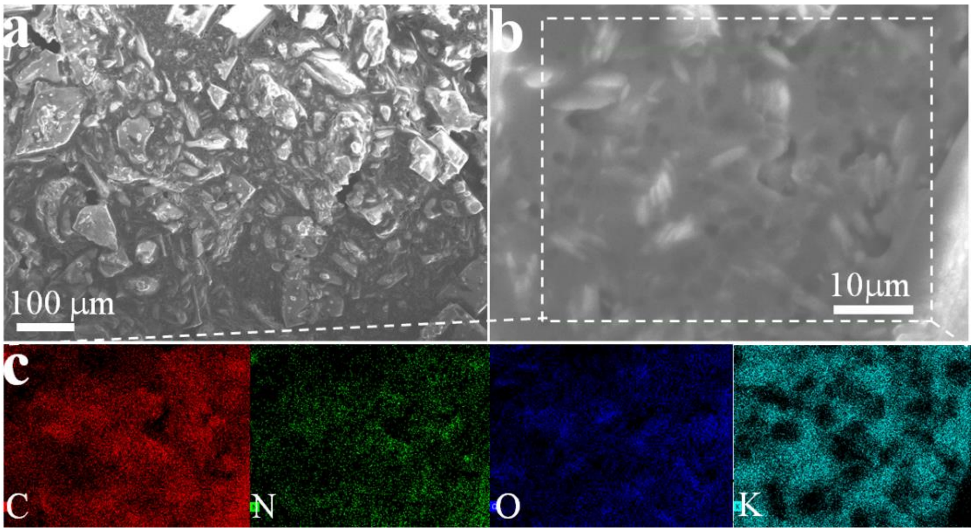

Fig. S2 SEM images (**a, b**) and the elemental maps (**c**) of the dried PBC/His mixture with a molar ratio of 2.0

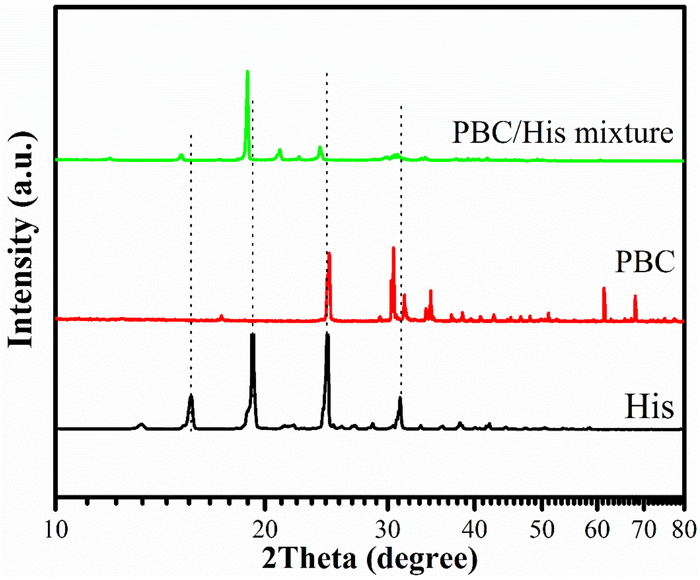

Fig. S3 Wide-angle XRD patterns of pure His, pure PBC and the dried PBC/His mixture with a molar ratio of 2.0. The diffraction peaks of the dried PBC/His mixture are totally different from those of pure PBC. In addition, compared with pure His, the most intensive diffraction peaks of the dried PBC/His mixture shift to lower angles and the peak intensity ratios are quite different. These results indicate the occurrence of acid-base reaction between His and PBC, probably forming a potassium salt of His.

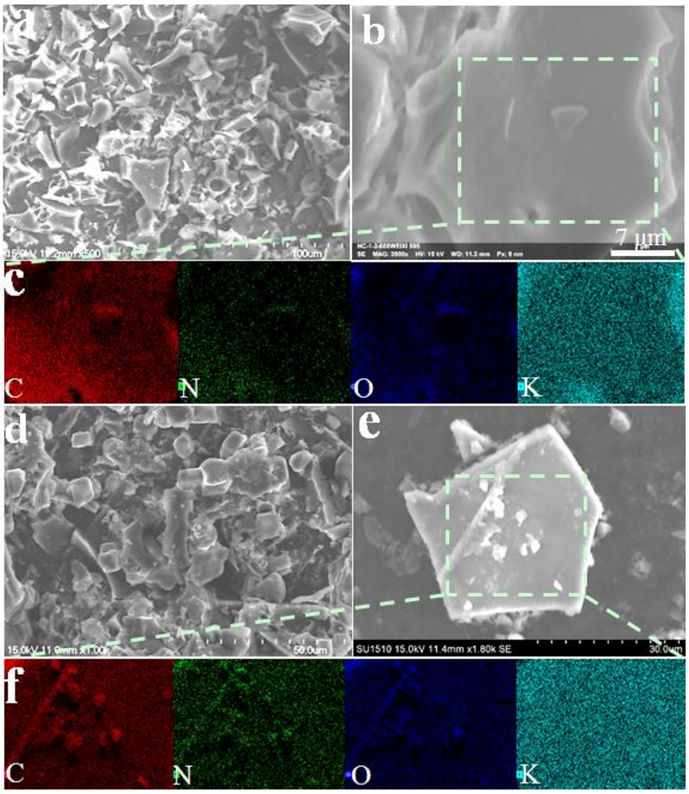

Fig. S4 SEM images (**a, b, d, e**) and the elemental maps (**c, f**) of the composites obtained with a PBC/His molar ratio of 2.0 at 600 (**a-c**) and 900 ºC (**d-f**)

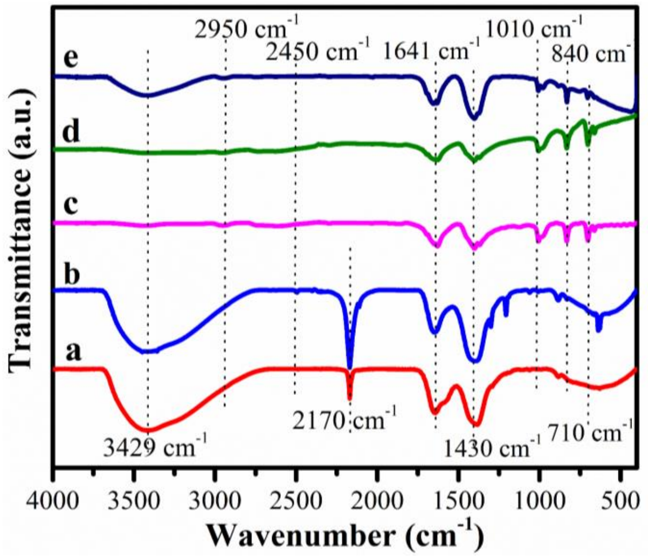

Fig. S5 FTIR spectra of the composites obtained with a PBC/His molar ratio of 2.0 at 400 (**a**), 600 (**b**), 700 (**c**), 800 (**d**), and 900 ºC (**e**)

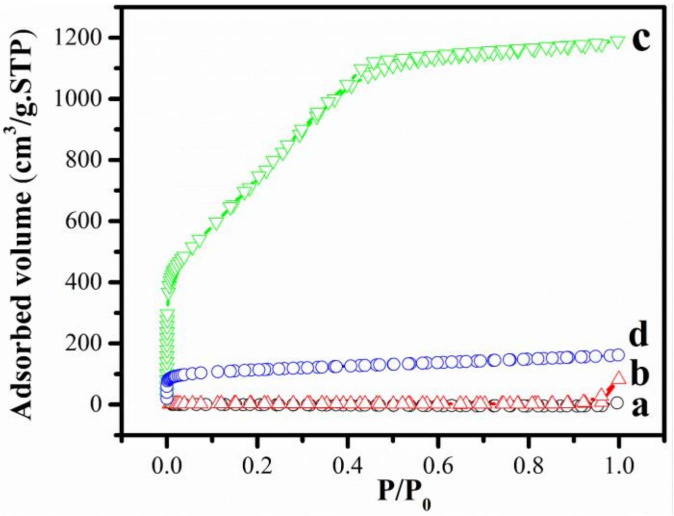

Fig. S6 N_2 sorption isotherms of the composites obtained with a PBC/His molar ratio of 2.0 at 600 (**a**) and 900 ºC (**b**), and the final HPNCF-2.0-900 sample (**c**), and the control sample (**d**) obtained by carbonizing pure His without the addition of PBC at 900 ºC

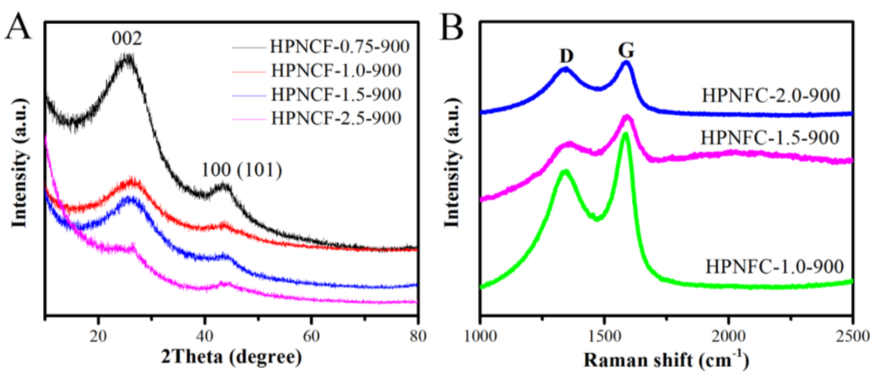

Fig. S7 Wide-angle XRD patterns (**A**) and Raman spectra (**B**) of the HPNCFs obtained at various PBC/His molar ratios at a fixed temperature of 900 ºC

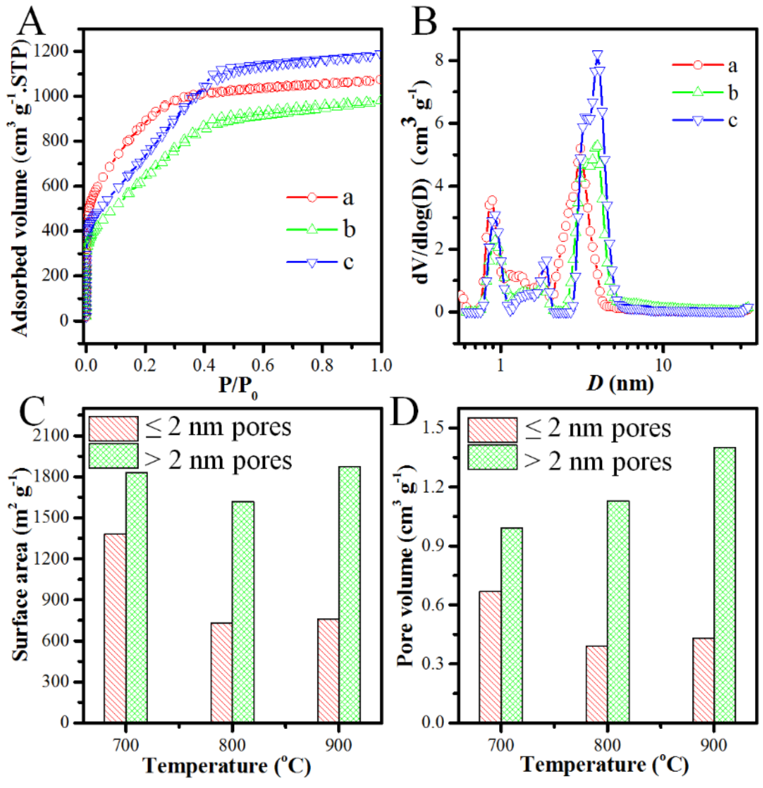

Fig. S8 N_2 sorption isotherms (**A**) and the PSD curves (**B**) of HPNCF-2.0-700 (**a**), HPNCF-2.0-800 (**b**), and HPNCF-2.0-900 (**c**). (**C**, **D**) are the corresponding pore-size-dependent surface area and pore volume variations with the increase of temperature at a fixed PBC/His molar ratio of 2.0

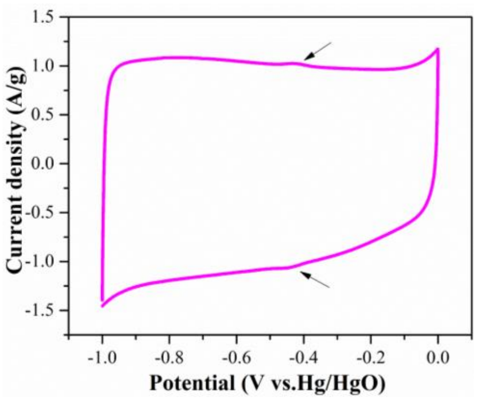

Fig. S9 Magnified CV curve of the electrode made of the sample HPNCF-2-900 at scan rate of 5 mV s^{-1}

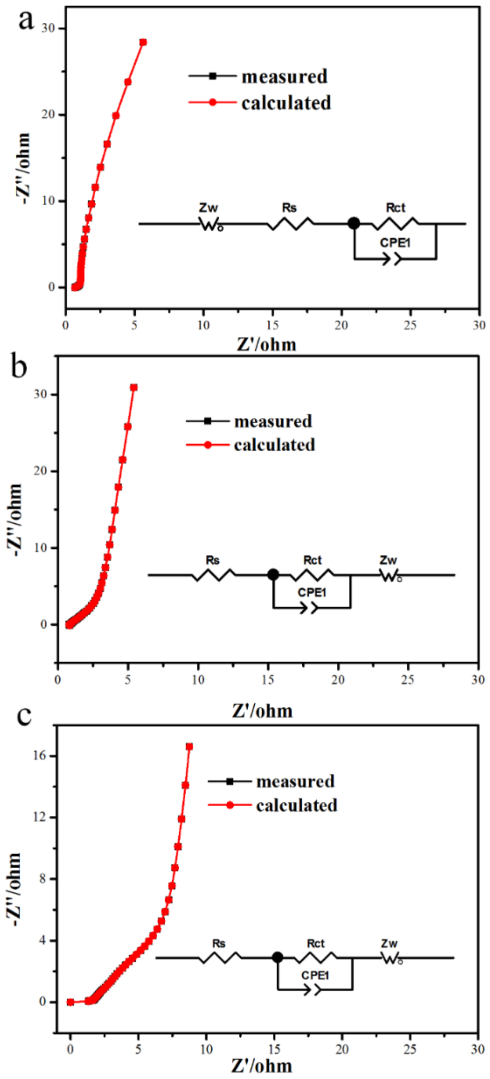

Fig. S10 The measured and calculated Nyquist plots, and the corresponding equivalent circuits of HPNCF-2.0-900 (**a**), HPNCF-2.0-800 (**b**), and HPNCF-2.0-700 (**c**)

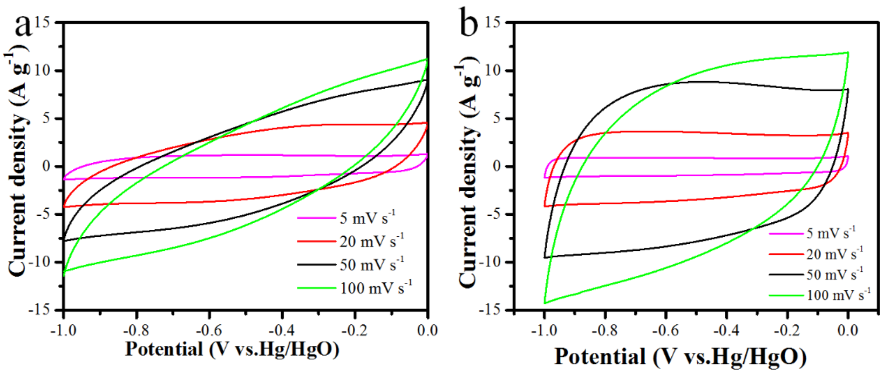

Fig. S11 CV curves of HPNCF-2.0-700 (**a**) and HPNCF-2.0-800 (**b**) at various scan rates

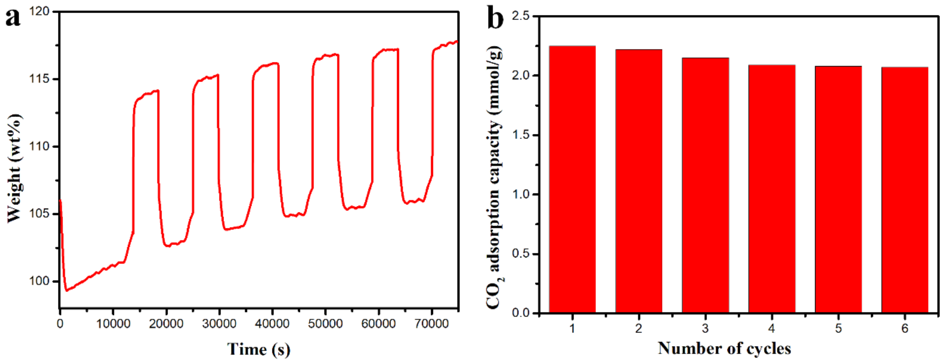

Fig. S12 The CO_2 adsorption/desorption cyclic performance for the sample HPNCF-1.0-700: weight change as a function of time (**a**) and the corresponding CO_2 adsorption capacity as a function of cycle number (**b**). The analysis was conducted in TG. The sample was first activated at 120 °C for 3 h under N_2, followed by adsorption in CO_2 atmosphere at 25 °C for 80 min and desorption at 120 °C in N_2 atmosphere for 30 min. The gas flow was 50 mL/min).

Table S1 A comparative summary of the physicochemical properties and performances in supercapacitors and CO_2 capture for a range of typical reported 3D hierarchically porous carbon materials in literature

Material	S_{BET} (m^2 g^{-1})	V_t (cm^3 g^{-1})	V_{mic} (cm^3 g^{-1})	N (wt%)	I (A g^{-1})	C_s (F g^{-1})	Electrolyte	Q_{CO2} [b] (mmol/g)	Refs.
2D-HPC	2406	/	/	9.4 [a]	0.5	280	6M KOH	/	[S1]
PGBC-1	1732	0.97	0.80	/	0.5	222	6M KOH	/	[S2]
PFNC	1648	0.89	/	3.90 [a]	1.0	260	2M KOH	/	[S3]
HLPC	2725	1.28	/	/	0.2	342	6M KOH	/	[S4]
HTC-JG-900	2838	1.23	/	0.69	1.0	220	6M KOH	5.35	[S5]
$HPGC_{0.6-700}$	1320	1.15	0.4	/	1.0	274	6M KOH	/	[S6]
hNCNC800	1794	/	/	7.90 [a]	1.0	313	6M KOH	/	[S7]
NCA-1000	2356	1.12	0.35	1.24	0.5	222	1M H_2SO_4	3.60	[S8]
H-NMC-2.5	537	0.47	0.17	13.10	0.2	227	6M KOH	2.8 [c]	[S9]

N-IOC-0.27-900	1365	1.01	0.30	4.67	0.5	222	6M KOH	/	[S10]
HP-CF	1175	0.57	/	12.0 [a]	2 mVs^{-1}	222	3M KOH	/	[S11]
KPAC-800	2988	1.76	0.71	/	1.0	306	6M KOH	/	[S12]
KCU-C 4-1-4	2096	3.0	/	/	2 mVs^{-1}	221	1M H_2SO_4	/	[S13]
K900	2064	1.41	0.28	/	2 mVs^{-1}	118	6M KOH	/	[S14]
K800	2435	1.1	0.62	/	2 mVs^{-1}	291	6M KOH	/	[S15]
CIRMOF-3-950	553	0.34	0.21	3.3	5 mVs^{-1}	239	1M H_2SO_4	/	[S16]
3D HPG	1810	1.22	0.38	/	0.5	305	6M KOH	/	[S17]
THPC	2870	2.19	/	7.7	0.5	318	6M KOH	/	[S18]
GHC-17	2818	1.32	1.02	/	1.0	462	6M KOH	/	[S19]
HN-DP-FSFC-2	2351	1.46	1.12	/	1.0	545	3M H_2SO_4	/	[S20]
PCC-2	1732	/	/	/	0.5	339	3M KOH	/	[S21]
$NPC_{0.5}$-700	425	/	/	/	1.0	180	6M KOH	/	[S22]
HPC-0.5	2544	2.02	0.89	/	1.0	261	6M KOH	/	[S23]
NPC-700	2846	/	/	2.83[a]	1.0	324	6M KOH	/	[S24]
HPNCF-2.5-900	2793	1.99	0.43	5.90	0.5	240	6M KOH	/	This
HPNCF-2.0-700	3209	1.66	0.67	14.52	/	/	/	4.13	work

[a]: The N content percentage is in atomic ratio, [b]: the CO_2 adsorption capacity at 273 K and 1 bar, [c]: the capacity at 298 K and 1 bar.

Supplementary References

[S1]L. Yao, Q. Wu, P. Zhang, J. Zhang, D. Wang et al., Scalable 2D hierarchical porous carbon nanosheets for flexible supercapacitors with ultrahigh energy density. Adv. Mater. **30**, 1706054 (2018). https://doi.org/10.1002/adma.201706054

[S2]Y. Gong, D. Li, C. Luo, Q. Fu, C. Pan, Highly porous graphitic biomass carbon as advanced electrode materials for supercapacitors. Green Chem. **19**, 4132-4140 (2017). https://doi.org/10.1039/C7GC01681F

[S3]G. Qu, S. Jia, H. Wang, F. Cao, L. Li et al., Asymmetric supercapacitor based on porous n-doped carbon derived from pomelo peel and NiO arrays. ACS Appl. Mater. Interfaces **8,** 20822-20830 (2016). https://doi.org/10.1021/acsami.6b06630

[S4]Q. Liang, L. Ye, Z. H. Huang, Q. Xu, Y, Bai, F. Kang, Q. H. Yang, A honeycomb-like porous carbon derived from pomelo peel for use in high-performance supercapacitors. Nanoscale **6**, 13831-13837 (2014). https://doi.org/10.1039/C4NR04541F

[S5]Y. Liu, B. Huang, X. Lin, Z. Xie, Biomass-derived hierarchical porous carbons: boosting the energy density of supercapacitors via an ionothermal approach. J. Mater. Chem. A **5**, 13009-13018 (2017). https://doi.org/10.1039/C7TA03639F

[S6]F. Ma, D. Ma, G. Wu, W. Geng, J. Shao, S. Song, J. Wan, J. Qiu, Construction of 3D nanostructure hierarchical porous graphitic carbons by charge-induced self-assembly and nanocrystal-assisted catalytic graphitization for supercapacitors. Chem. Commun. **52**, 6673-6676 (2016). https://doi.org/10.1039/C6CC02147F

[S7]J. Zhao, H. Lai, Z. Lyu, Y. Jiang, K. Xie et al., Hydrophilic hierarchical nitrogen-doped carbon nanocages for ultrahigh supercapacitive performance. Adv. Mater. **27**, 3541-3545 (2015). https://doi.org/10.1002/adma.201500945

[S8]Q. Du, T. Cheng, Y. Liu, N. Li, X. Wang, The use of natural hierarchical porous carbon from artemia cyst shells alleviates power decay in activated carbon air-cathode. Electrochim. Acta **315**, 41-47 (2019). https://doi.org/10.1016/j.electacta.2019.05.098

[S9]J. Wei, D. Zhou, Z. Sun, Y. Deng, Y. Xia, D. Zhao, A controllable synthesis of rich nitrogen-doped ordered mesoporous carbon for CO_2 capture and supercapacitors. Adv. Funct. Mater. **23**, 2322-2328 (2013). https://doi.org/10.1002/adfm.201202764

[S10] Y. Yao, Z. Chen, A. Zhang, J. Zhu, X. Wei et al., Surface-coating synthesis of nitrogen-doped inverse opal carbon materials with ultrathin micro/mesoporous graphene-like walls for oxygen reduction and supercapacitors. J.Mater. Chem. A **5**, 25237-25248 (2017). https://doi.org/10.1039/C7TA08354H

[S11] J. Chen, J. Xu, S. Zhou, N. Zhao,C.-P. Wong, Nitrogen-doped hierarchically porous carbon foam: A free-standing electrode and mechanical support for high-performance supercapacitors. Nano Energy **25,** 193-202 (2016). https://doi.org/10.1016/j.nanoen.2016.04.037

[S12] P. Cheng, S. Gao, P. Zang, X. Yang, Y. Bai, H. Xu, Z. Liu, Z. Lei, Hierarchically porous carbon by activation of shiitake mushroom for capacitive energy storage. Carbon **93**, 315-324 (2015). https://doi.org/10.1016/j.carbon.2015.05.056

[S13] L. Estevez, R. Dua, N. Bhandari, A. Ramanujapuram, P. Wang, E.P. Giannelis, A facile approach for the synthesis of monolithic hierarchical porous carbons – high performance materials for amine based CO_2 capture and supercapacitor electrode. Energy Environ. Sci. **6**, 1785-1790 (2013). https://doi.org/10.1039/C3EE40549D

[S14] P. Hao, Z. Zhao, J. Tian, H. Li, Y. Sang et al., Hierarchical porous carbon aerogel derived from bagasse for high performance supercapacitor electrode. Nanoscale **6**, 12120-12129 (2014). https://doi.org/10.1039/C4NR03574G

[S15] P. Hao, Z. Zhao, Y. Leng, J. Tian, Y. Sang et al., Graphene-based nitrogen self-doped hierarchical porous carbon aerogels derived from chitosan for high performance supercapacitors. Nano Energy **15**, 9-23 (2015). https://doi.org/10.1016/j.nanoen.2015.02.035

[S16] J.W. Jeon, R. Sharma, P. Meduri, B.W. Arey, H.T. Schaef et al., In situ one-step synthesis of hierarchical nitrogen-doped porous carbon for high-performance supercapacitors. ACS Appl. Mater. Interfaces **6**, 7214-7222 (2014). https://doi.org/10.1021/am500339x

[S17] Y. Li, Z. Li, P. K. Shen, Simultaneous formation of ultrahigh surface area and three-dimensional hierarchical porous graphene-like networks for fast and highly stable supercapacitors. Adv. Mater. **25**, 2474-2480 (2013). https://doi.org/10.1002/adma.201205332

[S18] L. Qie, W. Chen, H. Xu, X. Xiong, Y. Jiang et al., Synthesis of functionalized 3D hierarchical porous carbon for high-performance supercapacitors. Energy Environ. Sci. **6**. 2497-2504 (2013). https://doi.org/10.1039/C3EE41638K

[S19] Q. Zhang, K. Han, S. Li, M. Li, J. Li, K. Ren, Synthesis of garlic skin-derived 3D hierarchical porous carbon for high-performance supercapacitors. Nanoscale **10**, 2427-2437 (2018). https://doi.org/10.1039/C7NR07158B

[S20] C. Chang, M. Li, H. Wang, S. Wang, X. Liu, H. Liu, L. Li, A novel fabrication strategy for doped hierarchical porous biomass-derived carbon with high microporosity for ultrahigh-capacitance supercapacitors. J. Mater. Chem. A **7**, 19939-19949 (2019). https://doi.org/10.1039/C9TA06210F

[S21] Y. Liu, M. Zhang, L. Wang, Y. Hou, C. Guo, H. Xin, S. Xu, A biomass carbon material with microtubule bundling and natural O-doping derived from goldenberry calyx and its electrochemical performance in supercapacitor. Chin. Chem. Lett. (2019). https://doi.org/10.1016/j.cclet.2019.05.045

[S22] C. Xuan, Z. Peng, J. Wang, W. Lei, K. Xia, Z. Wu, W. Xiao, D. Wang, Biomass derived nitrogen doped carbon with porous architecture as efficient electrode materials for supercapacitors. Chin. Chem. Lett. **28**, 2227-2230 (2017). https://doi.org/10.1016/j.cclet.2017.09.009

[S23] L. Miao, X. Qian, D. Zhu, T. Chen, G. Ping, Y. Lv, W. Xiong, Y. Liu, L. Gan, M. Liu, From interpenetrating polymer networks to hierarchical porous carbons for advanced supercapacitor electrodes. Chin. Chem. Lett. **30**, 1445-1449 (2019). https://doi.org/10.1016/j.cclet.2019.03.010

[S24] S. Sun, F. Han, X. Wu, Z. Fan, One-Step Synthesis of Biomass Derived O, N-codoped hierarchical porous carbon with high surface area for supercapacitors. Chin. Chem. Lett. (2019). https://doi.org/10.1016/j.cclet.2019.11.023
